# *Plasmodium falciparum* selectively degrades α-spectrin of infected erythrocytes after invasion

**DOI:** 10.1128/mbio.03510-23

**Published:** 2024-03-12

**Authors:** Kexin Zheng, Qilong Li, Ning Jiang, Yanxin Zhang, Yuxin Zheng, Yiwei Zhang, Ying Feng, Ran Chen, Xiaoyu Sang, Qijun Chen

**Affiliations:** 1 Key Laboratory of Livestock Infectious Diseases, Ministry of Education, College of Animal Science and Veterinary Medicine, Shenyang Agricultural University, Shenyang, China; 2 Key Laboratory of Ruminant Infectious Disease Prevention and Control (East), Ministry of Agriculture and Rural Affairs, College of Animal Science and Veterinary Medicine, Shenyang Agricultural University, Shenyang, China; 3 Research Unit for Pathogenic Mechanisms of Zoonotic Parasites, Chinese Academy of Medical Sciences, Shenyang, China; 4 Engineering Research Center of Food Fermentation Technology, College of Food Science, Shenyang Agricultural University, Shenyang, China; University of Geneva, Geneva, Switzerland

**Keywords:** *Plasmodium falciparum*, α-spectrin, PfPI3K, ubiquitination, erythrocyte

## Abstract

Remodeling the erythrocyte membrane and skeleton by the malarial parasite *Plasmodium falciparum* is closely associated with intraerythrocytic development. However, the mechanisms underlying this association remain unclear. In this study, we present evidence that erythrocytic α-spectrin, but not β-spectrin, was dynamically ubiquitinated and progressively degraded during the intraerythrocytic development of *P. falciparum,* from the ring to the schizont stage. We further observed an upregulated expression of *P. falciparum* phosphatidylinositol 3-kinase (PfPI3K) in the infected red blood cells during the intraerythrocytic development of the parasite. The data indicated that PfPI3K phosphorylated and activated erythrocytic ubiquitin-protein ligase, leading to increased α-spectrin ubiquitination and degradation during *P. falciparum* development. We further revealed that inhibition of the activity of PfPI3K impaired *P. falciparum* development *in vitro* and *Plasmodium berghei* infectivity in mice. These findings collectively unveil an important mechanism of PfPI3K-ubiquitin-mediated degradation of α-spectrin during the intraerythrocytic development of *Plasmodium* species. Proteins in the PfPI3K regulatory pathway are novel targets for effective treatment of severe malaria.

**IMPORTANCE:**

*Plasmodium falciparum* is the causative agent of severe malaria that causes millions of deaths globally. The parasite invades human red blood cells and induces a cascade of alterations in erythrocytes for development and proliferation. Remodeling the host erythrocytic cytoskeleton is a necessary process during parasitization, but its regulatory mechanisms remain to be elucidated. In this study, we observed that erythrocytic α-spectrin is selectively degraded after *P. falciparum* invasion, while β-spectrin remained intact. We found that the α-spectrin chain was profoundly ubiquitinated by E3 ubiquitin ligase and degraded by the 26S proteasome. E3 ubiquitin ligase activity was regulated by *P. falciparum* phosphatidylinositol 3-kinase (PfPI3K) signaling. Additionally, blocking the PfPI3K-ubiquitin-proteasome pathway in *P. falciparum*-infected red blood cells reduced parasite proliferation and infectivity. This study deepens our understanding of the regulatory mechanisms of host and malarial parasite interactions and paves the way for the exploration of novel antimalarial drugs.

## INTRODUCTION

*Plasmodium falciparum* exports proteins to interact with the erythrocyte membrane and skeleton (EMS), contributing to malaria pathogenesis (1). Remodeling of the EMS changes the erythrocyte shape from biconcave to spherical, affecting intracellular development, proliferation, and transportation of nutrients and metabolites (2, 3). Spectrin, a major component of the EMS, is a long, flexible, worm-like protein with α- and β-spectrin as the main components (4–6). The erythrocyte cytoskeleton is primarily composed of two complexes, the ankyrin complex and the actin junctional complex, which serve as scaffolds for a network of α/β-spectrin heterodimers that contribute to the shape and deformability of erythrocytes (7–10). *P. falciparum* erythrocyte membrane protein 3 has been reported to bind α-spectrin at the actin junctional complex, leading to destabilization of the network, which regulates the egress of *P. falciparum* from infected red blood cells (iRBCs) (2, 11). Previous studies have indicated that the ring-infected erythrocyte surface antigen released from *P. falciparum* also increases the pore size of spectrin meshes, facilitating parasites’ development (12). However, the exact molecular mechanisms of α-spectrin destabilization are still unknown.

The interplay between phosphorylation and ubiquitin-mediated degradation is common in spectrin degradation in reticulocytes, whereas mature RBCs lack the 26S proteasome (12, 13). Upon *P. falciparum* infection, the *Pf*-derived 20S proteasome can degrade non-ubiquitinated RBC skeleton proteins (14, 15), suggesting that the 20S proteasome plays an important role in the degradation of non-ubiquitinated EMS during parasite invasion; however, the degradation of ubiquitinated EMS remains unclear. Our previous study revealed that *P. falciparum* iRBCs were enriched with the 26S proteasome, compared to uninfected RBCs (16). This raises the possibility of a *Pf*-derived 26S proteasome-dependent mechanism for the degradation of the ubiquitinated host cytoskeleton. However, this hypothesis warrants further investigation.

The phosphatidylinositol 3-kinase (PI3K) signaling pathway regulates the activation of the ubiquitin-26S proteasome in eukaryotic cells except mature RBCs (17). *P. falciparum* parasites possess a complete PI3K (PfPI3K) signaling pathway (17–20), which is in accordance with our previous study that the PfPI3K signaling pathway was enriched in ring-stage *P. falciparum*, compared to that in RBCs (16), suggesting PfPI3K signaling might play an important role in the intraerythrocytic development of *P. falciparum*. However, the specific roles and mechanisms of action remain unclear.

In this study, we revealed that the *P. falciparum*-derived PI3K phosphorylates and activates the ubiquitin-protein ligase and regulates the secretion effect of the 26S proteasome from the parasite to the erythrocyte cytoplasm, leading to increased ubiquitination of α-spectrin and its degradation during its intraerythrocytic development. Moreover, treatment with a PfPI3K inhibitor reduced *P. falciparum* development *in vitro* and *Plasmodium berghei* infectivity in mice. Overall, this study revealed a novel mechanism by which intracellular parasites reshape host cells to facilitate their development and proliferation.

## RESULTS

### Erythrocytic α-spectrin was progressively degraded during *P. falciparum* development

**Fig 1:**
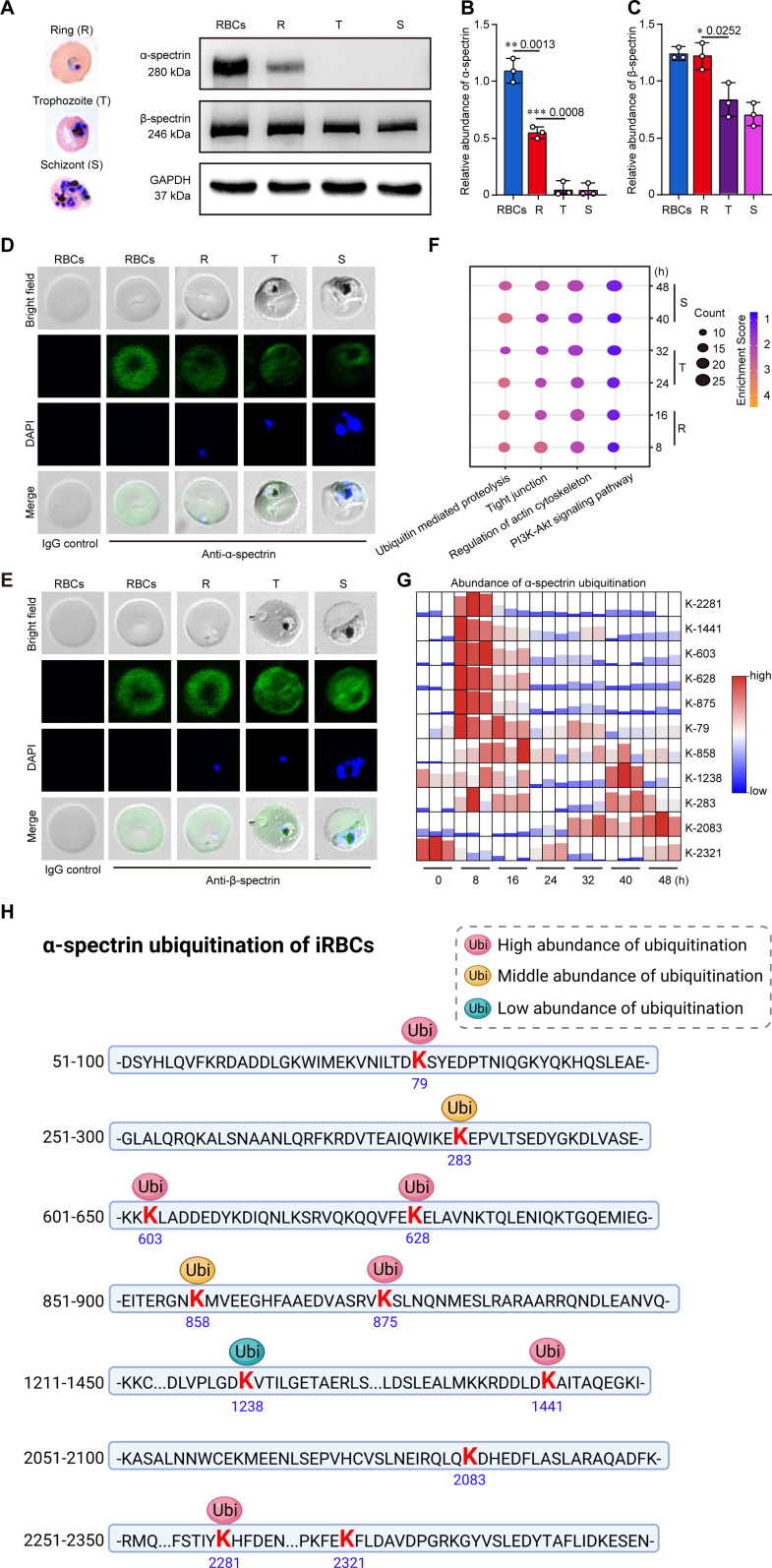
α-Spectrin is mainly degraded in the RBCs after *P. falciparum* invasion. (**A**) Western blots of α-spectrin and β-spectrin in *P. falciparum*-iRBCs at different erythrocytic stages. R, T, and S indicate *P. falciparum* at the ring, trophozoite, and schizont stages, respectively. (**B and C**) Relative quantification of α-spectrin and β-spectrin protein levels obtained in panel A. (**D and E**) α-Spectrin and β-spectrin in iRBCs were detected via immunofluorescence staining. (**F**) The bubble chart represents the enrichment of *Plasmodium* proteins in KEGG pathways over 8-, 16-, 24-, 32-, 40-, and 48-h post-parasite invasion. The size of the circles indicates the number of proteins enriched in the signaling pathway, and the color of each circle indicates the enrichment score. The enrichment score was calculated by comparing the proteins’ enrichment in iRBCs to that in the RBCs, with blue representing the lowest score and orange the highest score. (**G**) The heatmap represents the abundance of α-spectrin ubiquitinated sites in iRBCs over 8-, 16-, 24-, 32-, 40-, and 48-h post-parasite invasion. Only the lysine 2321 in α-spectrin was ubiquitinated in uninfected RBC, nine more lysine residues were ubiquitinated after parasite invasion. (**H**) The distribution of ubiquitinated lysine residues in α-spectrin of *P. falciparum*-iRBCs is graphically represented.

*P. falciparum* modifies the host erythrocyte cytoskeleton during its development (2). First, we compared the abundance of stage-related alterations in the cytoskeleton of *P. falciparum*-iRBCs using western blotting. The abundance of α-spectrin in iRBCs at the ring stage was approximately 50% less than that of the uninfected RBCs (Fig. 1A and B), which decreased at the trophozoite stage to more than 60% of that in the ring stage (*P* < 0.05). There was a significant decrease from the early trophozoite to the schizont stages of the parasites (Fig. S1). However, no significant quantitative changes in β-spectrin were observed between the RBC infected with ring stage parasite and the uninfected RBCs. The decreases in the abundance of β-spectrin in iRBCs were observed in the trophozoite and schizont stages, which were approximately 10% less than that of the ring stage (Fig. 1A and C), indicating the processing of the two components was regulated by different mechanisms. The results were further confirmed via immunofluorescence assays (Fig. 1D and E). These data suggest that α-spectrin is the primary degradation target during *P. falciparum* development.

To further elucidate the molecular mechanisms underlying this phenomenon, we analyzed the expression of proteins in different signaling pathways in uninfected RBCs and iRBCs at different developmental stages of *P. falciparum* using liquid chromatography-tandem mass spectrometry (LC-MS/MS). We found that proteins of *Plasmodium* in the PI3K-Akt pathway were predominantly enriched (Fig. 1F). We further identified 11 ubiquitination sites on α-spectrin that occurred predominantly at the ring stage of the parasite, but ubiquitination on β-spectrin was not observed. Of the 11 ubiquitination sites on α-spectrin, six ubiquitination sites were upregulated compared with uninfected RBC controls (Fig. 1G and H; Table S2). These data suggest that the selective degradation of α-spectrin, but not β-spectrin, is associated with the ubiquitination and PI3K signaling pathways.

### Inhibition of PfPI3K activity impaired *P. falciparum* development and *P. berghei* in mice

**Fig 2:**
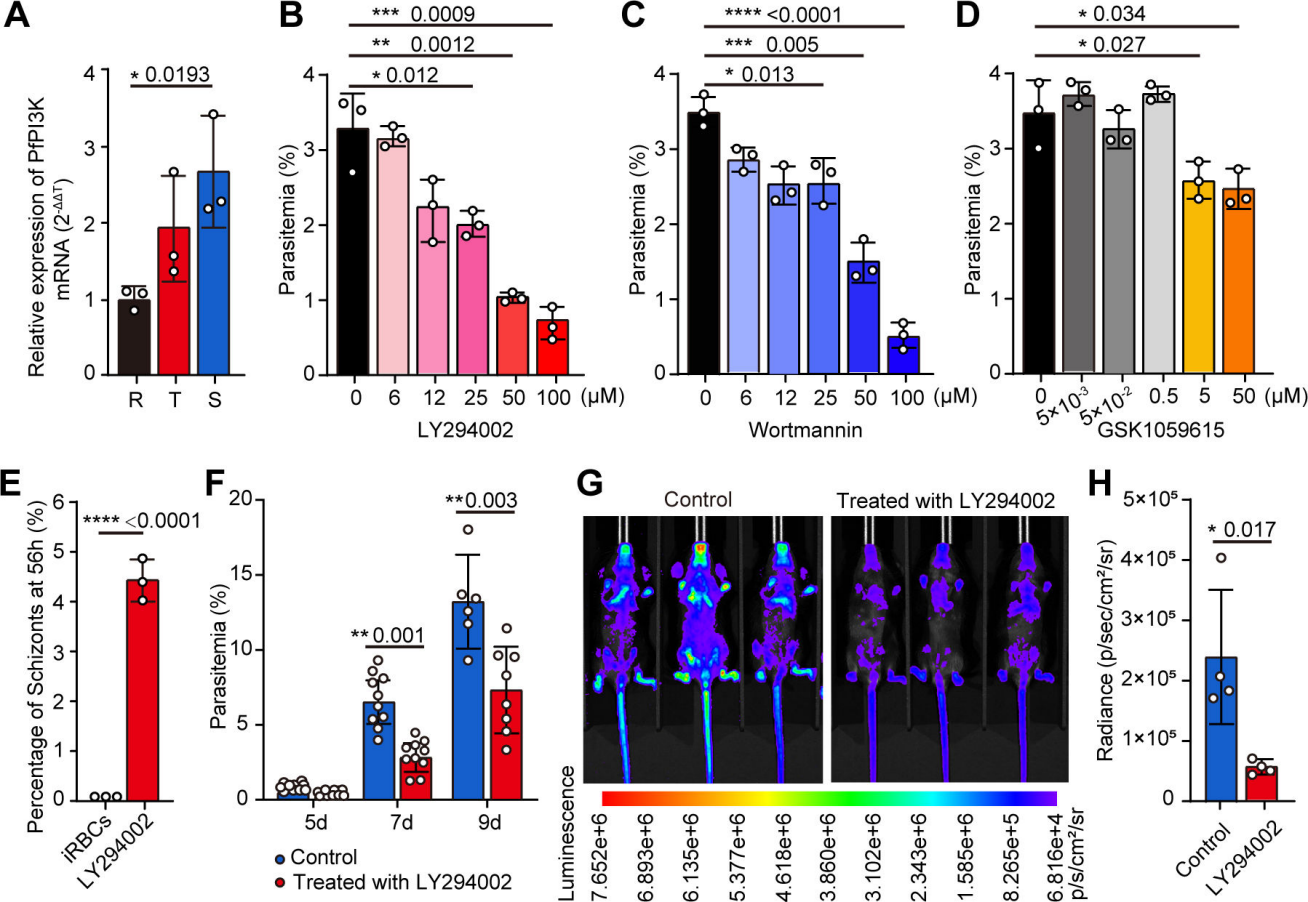
PfPI3K is essential for blood-stage *Plasmodium* parasite infection. (**A**) *PfPI3K* transcription in ring, trophozoite, and schizont stages of *P. falciparum* 3D7 parasites significantly increased with their development, as shown via quantitative qRT-PCR. (**B–D**) *P. falciparum* 3D7 parasitemia under treatment with PfPI3K inhibitors LY294002, wortmannin, and GSK1059615. LY294002 and wortmannin showed a dose-dependent inhibitory effect on parasite proliferation, whereas the effect of GSK1059615 was not significant. (**E**) *P. falciparum* 3D7 parasitemia under treatment with PfPI3K inhibitor LY294002 (50 µM) showed an inhibitory effect on parasite egression from the infected RBCs. (**F and G**) LY294002 treatment significantly reduced parasitemia in *P. berghei* ANKA-infected mice in a period of 9 days. (**H**) Statistical analysis of the luminescence showed that LY294002 treatment significantly reduced parasitemia in *P. berghei* ANKA-infected mice.

PI3K is a crucial regulator of erythropoiesis (19, 20). However, mature blood cells, which are essential hosts for *P. falciparum*, lack the complete PI3K pathway. Previous studies showed that PfPI3K is required for *P. falciparum* development and survival (18, 20). Therefore, the precise function of this pathway during *P. falciparum* infection requires further investigation. *PfPI3K* expression was assessed using quantitative RT-PCR (qRT-PCR) in *P. falciparum* 3D7 parasites at the ring, trophozoite, and schizont stages. The *PfPI3K* transcript levels gradually increased during *P. falciparum* development (Fig. 2A). We also found that PfPI3K was predominantly located in the parasite but also secreted to the RBC cytoplasm (Fig. S2A and B).

To explore the function of PfPI3K, we assessed parasitemia in the context of PfPI3K deficiency. Blocking PfPI3K of the ring-stage parasite with three PI3K-specific inhibitors, LY294002, wortmannin, and GSK1059615, significantly inhibited parasite proliferation in a dose-dependent manner compared to the control medium. This inhibition was detected at 72 h (Fig. 2B through D; Fig. S2C). Blocking PfPI3K of the ring-stage parasite with LY294002 (50 µM), resulted in a significantly higher percentage of schizont-stage parasites (at 56 h) compared to the iRBC control (Fig. 2E), indicating that LY294002 inhibited parasites egression from the infected RBCs. We next examined the inhibitory effect of LY294002 on the *in vivo* proliferation of the rodent parasite *P. berghei* ANKA in mice and found that treatment with LY294002 resulted in significantly reduced parasitemia in the mice (Fig. 2F through H; Fig. S2D), which were consistent with the *in vitro* experiments with the *P. falciparum* parasites (Fig. 2B). Thus, our data indicate that PfPI3K is essential for *P. falciparum* development.

### PfPI3K was associated with erythrocytic α-spectrin degradation

**Fig 3:**
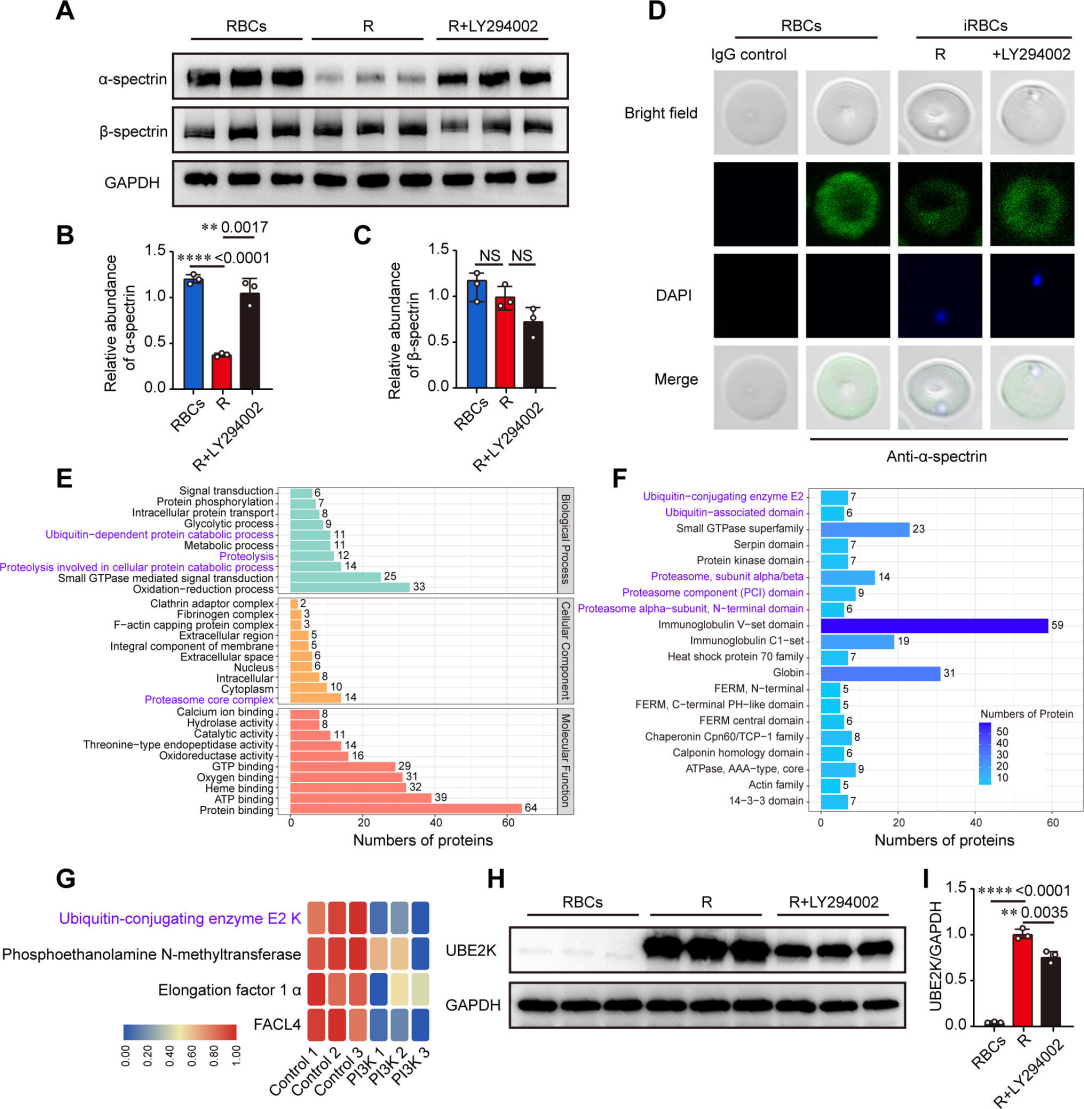
PfPI3K regulates *P. falciparum* ubiquitin-proteasome system. (**A**) Western blots of α-spectrin and β-spectrin in the ring-stage *P. falciparum*-iRBCs with or without the treatment of the PfPI3K inhibitor LY294002 (50 µM). (**B and C**) Relative quantification of α-spectrin and β-spectrin protein levels shown in panel A. (**D**) Immunofluorescence staining of α-spectrin with a protein-specific antibody in cells with or without LY294002 (50 µM) treatment. (**E**) Functional categorization of proteins identified in iRBC of ring-stage parasites by proteomic analysis after treatment with LY294002. (**F**) Categorization of identified proteins based on structural domains in proteins identified in iRBC of ring-stage parasites after treatment with LY294002. Proteins associated with ubiquination and proteasome are highlighted in blue color. (**G**) Downregulated proteins were identified in iRBC of ring-stage parasites by proteomic analysis after treatment with LY294002. Ubiquitin-conjugating enzymes E2 K (UBE2K) were the proteins predominantly affected by the inhibitor. (**H**) Western blots of UBE2K in ring-stage *P. falciparum*-iRBCs with or without the treatment of the PfPI3K inhibitor LY294002 (50 µM). (**I**) Relative quantification of UBE2K protein level shown in panel H. Inhibition of PfPI3K activity significantly downregulated the UBE2K.

To determine the role of PfPI3K in α-spectrin degradation during parasite development, we examined the abundance of α-spectrin in iRBCs infected with ring-stage *P. falciparum* treated with the PI3K inhibitor LY294002. Significant inhibition of α-spectrin degradation was observed in LY294002-treated cells (Fig. 3A and B). However, LY294002 had no effect on β-spectrin abundance (Fig. 3A and C). Immunofluorescence staining with α-spectrin-specific antibodies was consistent with the western blot results (Fig. 3D).

Using a quantitative proteomic experiment, we found that PfPI3K was involved in the regulation of the proteasome core complex pathway and ubiquitin-conjugating enzyme E2 (Fig. 3E and F; Fig. S3 and 4). Inhibition of PfPI3K activity downregulated the ubiquitin-conjugating enzyme E2 K (Fig. 3G through I), suggesting that PfPI3K contributes to the activation of the ubiquitin-proteasome pathway, which is likely associated with α-spectrin processing.

### PfPI3K regulated α-spectrin ubiquitination and degradation

**Fig 4:**
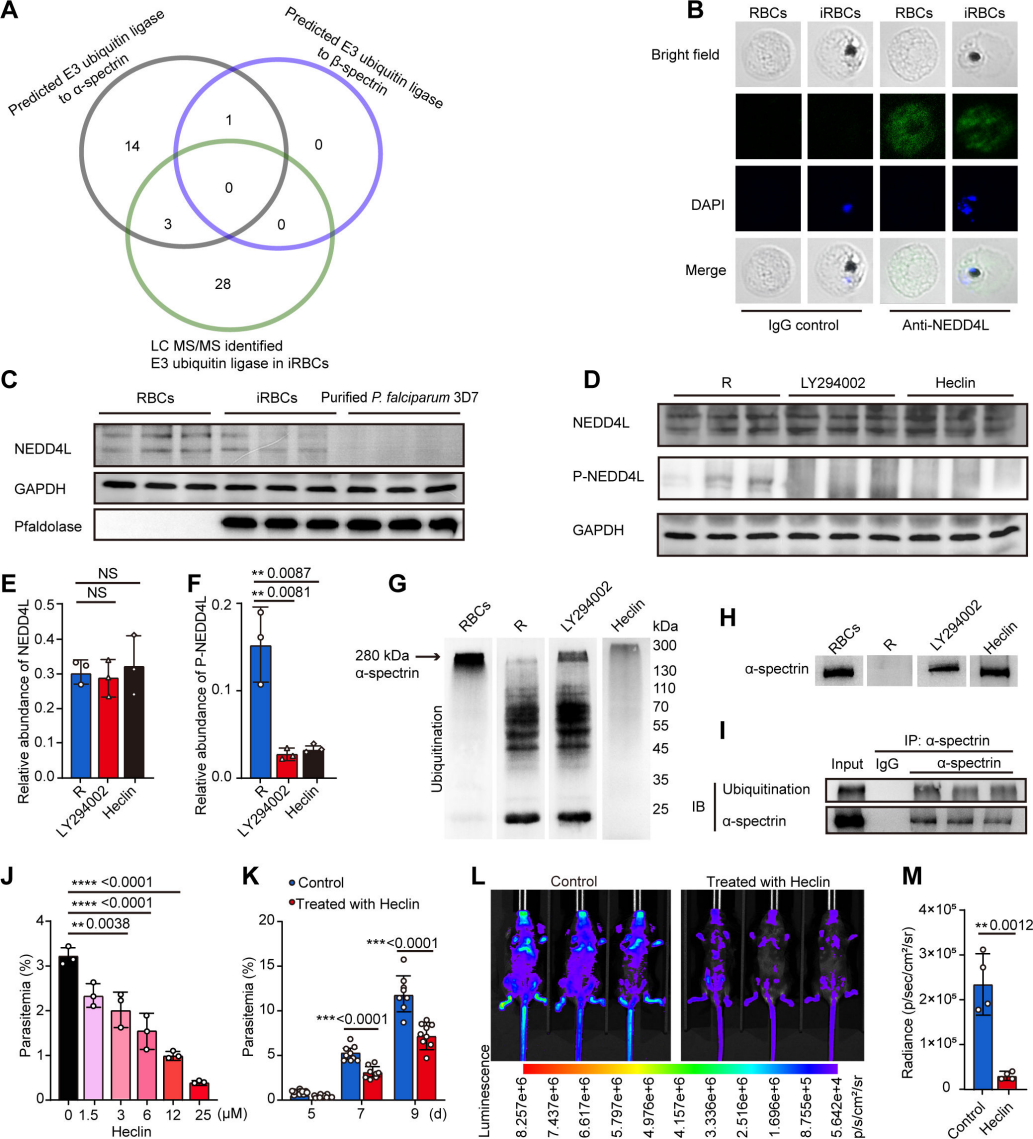
Host NEDD4L is necessary for the ubiquitination of α-spectrin in the blood stage of *Plasmodium* parasites. (**A**) Of the 18 E3 ubiquitin ligases potentially involved in the interaction with α-spectrin, 3 were identified in the proteomic analysis, and none were involved in β-spectrin ubiquitination. The black and blue circles represent the predicted E3 ubiquitin ligase to α-spectrin and β-spectrin, respectively (http://ubibrowser.bio-it.cn/); The green circle represents the E3 ubiquitin ligase identified by LC-MS/MS in iRBCs. (**B**) NEDD4L was detected via immunofluorescence staining in RBCs and iRBCs with an anti-NEDD4L antibody. (**C**) Western blots of NEDD4L in whole-cell extracts from RBCs, iRBCs, and purified *P. falciparum* 3D7 with an anti-NEDD4L antibody. NEDD4L was detected in both RBCs and iRBCs but not in the purified *P. falciparum*. (**D**) Western blots of NEDD4L and P-NEDD4L with anti-NEDD4L and anti-P-NEDD4L antibodies in the ring stage of *P. falciparum*-iRBCs after treatment with the PfPI3K and E3 ubiquitin ligase inhibitors LY294002 (50 µM) and heclin (5 µM), respectively, using e-BLOT touch imager. (**E and F**) Relative quantification of the NEDD4L and P-NEDD4L protein levels shown in panel D. (**G and H**) The ubiquitinated α-spectrin could not be degraded in RBCs. However, it was completely degraded in iRBCs with ring-stage parasites. The PfPI3K inhibitor LY294002 (50 µM) partially inhibited ubiquitination and degradation of α-spectrin, but the effect of NEDD4L inhibitor heclin (5 µM) was more significant. (**I**) RBC lysates were subjected to co-immunoprecipitation (IP) using anti-α-spectrin antibodies, and α-spectrin-ubiquitin complexes were detected with anti-ubiquitin antibody immunoblotting. Ubiquitination was observed on α-spectrin in RBC lysates (input). The α-spectrin-ubiquitin complex was not detected in the IP with an irrelevant IgG control. (**J**) Heclin showed significant inhibition of parasite proliferation in a dose-dependent manner, compared to the control medium. (**K and L**) Heclin treatment significantly reduced parasitemia in *P. berghei* ANKA-infected mice. The control mice were the same as that illustrated in Fig. 5L. (**M**) Statistical analysis of the luminescence showed that heclin treatment significantly reduced parasitemia in *P. berghei* ANKA-infected mice.

In most eukaryotic cells, protein stability is determined by the ubiquitination recognized by the proteasome complex, which is regulated by PI3K (21–24). To investigate the possible involvement of PfPI3K in α-spectrin ubiquitination of iRBCs, we performed a combined analysis using bioinformatics prediction of E3 ubiquitin ligases that interact with α-spectrin, followed by proteomic identification of E3 ligases in iRBCs. Eighteen E3 ubiquitin ligases were bioinformatically predicted, only one of which was predicted for β-spectrin ubiquitination. However, only three E3 ligases likely involved in α-spectrin ubiquitination were identified in the proteomic analysis, and a non-E3 ubiquitin ligase potentially involved in β-spectrin ubiquitination was also identified (Fig. 4A; Fig. S5A through D). Of the three enzymes, only E3 ubiquitin ligase (NEDD4L) was found localized in the cytoplasm of both RBC and iRBC (Fig. 4B and C), which showed detectable phosphorylation (Tables S3 and S4; Fig. S5D). Thus, we propose that NEDD4L containing a HECT domain was responsible for α-spectrin ubiquitination.

To elucidate whether PfPI3K regulated the expression or activation of E3 ligase, we quantified both E3 ligase and its phosphorylated form (p-E3 ligase, activated state) using western blot analysis. PfPI3K inhibition significantly suppressed the phosphorylation of the E3 ubiquitin-protein ligase NEDD4L but did not impair its expression (Fig. 4D through F). As the HECT domain of NEDD4L is essential for ubiquitination (25), we, therefore, examined the levels of ubiquitination and degradation of α-spectrin in the presence of the NEDD4L HECT domain inhibitor heclin. Inhibition of the HECT domain markedly suppressed α-spectrin ubiquitination (Fig. 4G and H). Next, we used a co-immunoprecipitation (IP) assay with anti-α-spectrin and anti-ubiquitin antibodies to determine whether α-spectrin was ubiquitinated. The results indicated that α-spectrin was polyubiquitinated (Fig. 4I). These findings suggest that the HECT domain of NEDD4L is involved in α-spectrin ubiquitination. In addition, treatment with the HECT domain inhibitor heclin significantly inhibited *P. falciparum* proliferation *in vitro* (Fig. 4J), parasite egression from the infected RBCs (Fig. S5E), and *P. berghei* ANKA infectivity in mice (Fig. 4K through M). These observations demonstrated that the HECT domain of NEDD4L is involved in dynamic ubiquitination and parasite development.

### Host α-spectrin was degraded by the PfPI3K-regulated ubiquitin protease pathway

**Fig 5:**
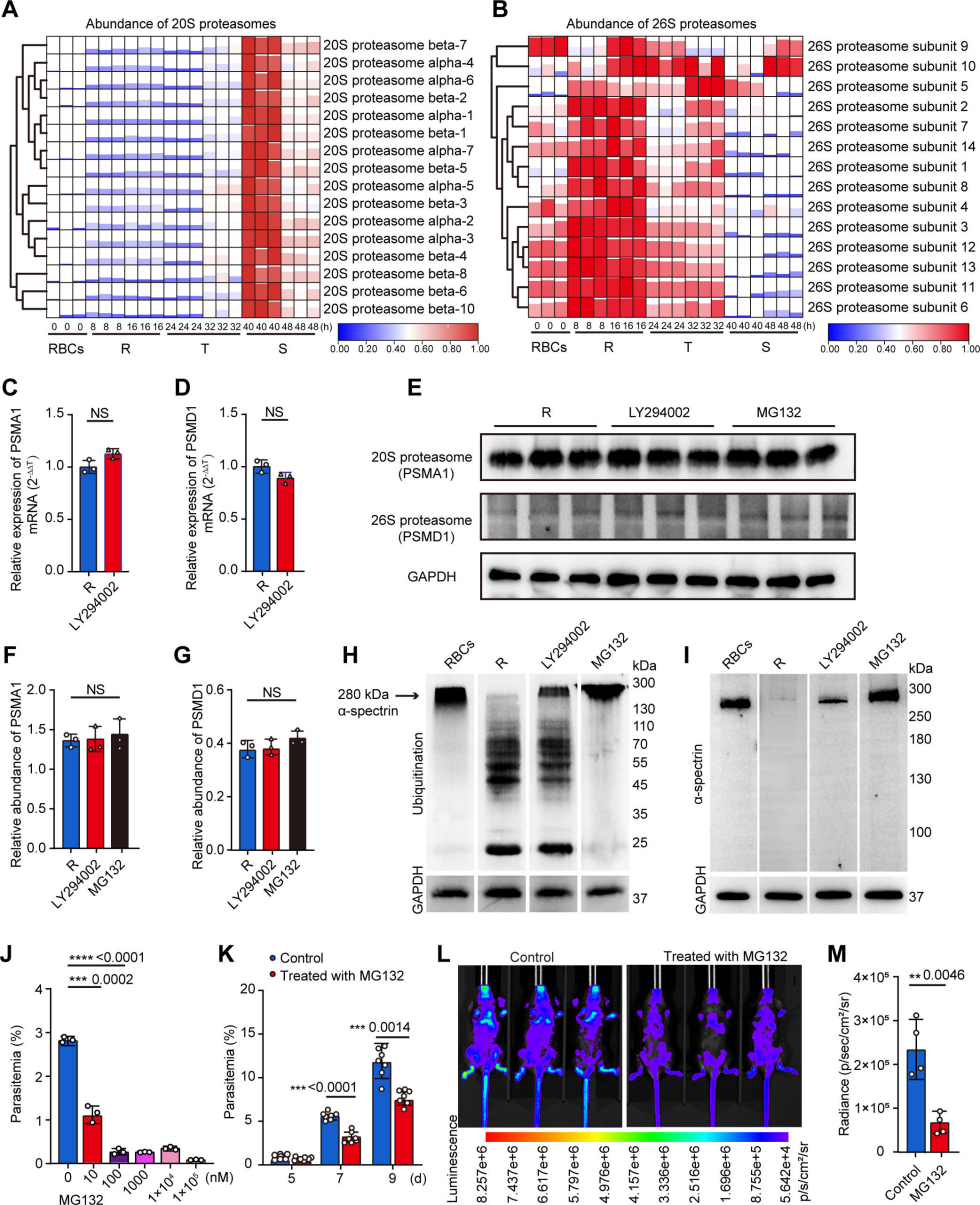
The 26S proteasome was involved in α-spectrin degradation at the early erythrocytic stage *P. falciparum*. (**A and B**) The heatmaps represent the abundance of the 20S (**A**) and 26S (**B**) proteasomes in the iRBCs over 8-, 16-, 24-, 32-, 40-, and 48-h post-parasite invasion. (**C and D**) Treatment with the PfPI3K and 26S proteasome inhibitors, LY294002 and MG132, did not alter PSMA1 (20S proteasome) and PSMD1 (26S proteasome) transcription in ring-stage *P. falciparum*. (**E**) Western blots show that treatment with the respective PfPI3K and 26S proteasome inhibitors, LY294002 (50 µM) and MG132 (100 nM), did not affect PSMA1 and PSMD1 expression. (**F and G**) Relative quantification of PSMA1 and PSMD1 protein levels shown in panel E. (**H and I**) The ubiquitinated α-spectrin completely degraded iRBCs with ring-stage parasites (R). PfPI3K inhibitor LY294002 (50 µM) partially inhibited the ubiquitination and degradation of α-spectrin, but the 26S proteasome inhibitor MG132 (100 nM) only inhibited the degradation of α-spectrin in iRBCs. (**J**) MG132 significantly inhibited *P. falciparum* proliferation *in vitro* in a dose-dependent manner, compared to the medium control. (**K and L**) MG132 treatment significantly reduced parasitemia in *P. berghei* ANKA-infected mice. The experiment was carried out continuously with the same group of mice injected with a saline solution (Fig. 4L, control). (**M**) Statistical analysis of the luminescence showed that MG132 treatment significantly reduced parasitemia in *P. berghei* ANKA-infected mice.

Previous studies indicated that 20S proteasome was involved in cytoskeletal network remodeling, which facilitated malarial parasite proliferation (14). However, the involvement of the 26S proteasome has not been clear. Here, we examined the abundance of the 20S and 26S proteasomes in different intraerythrocytic stages of *P. falciparum* using LC-MS/MS. The 20S and 26S proteasomes showed differential expression patterns in iRBCs with distinct peak points. Expression of the 20S proteasome peaked at the late trophozoite and schizont stages, whereas that of the 26S proteasome peaked at the early stage of the parasite, suggesting a necessary role of the 26S proteasome in the early stage of parasite development (Fig. 5A and B; Tables S5 and S6).

To determine whether PfPI3K regulates the degradation of ubiquitinated α-spectrin mediated via the 26S proteasome, we first examined whether the 26S proteasome is expressed in the context of PfPI3K deficiency. Inhibition of PfPI3K activity did not affect the mRNA or protein levels of the 26S proteasome in *P. falciparum* (Fig. 5C through G; Fig. S6A) but suppressed the secretion of 26S proteasome from the parasite to the erythrocyte cytoplasm (Fig. S6B). We next investigated the effect of a specific inhibitor 26S proteasome, MG132, on α-spectrin degradation. As expected, MG132 did not affect the expression of 26S proteasome represented by PSMD1 (Fig. 5E through G) but significantly inhibited α-spectrin degradation (Fig. 5H and I). Importantly, blocking 26S proteasome activity completely inhibited α-spectrin degradation. These observations clearly demonstrated that the 26S proteasome is predominantly expressed in the early parasite stages, which are critically involved in α-spectrin processing. In addition, treatment with MG132 significantly inhibited *P. falciparum* proliferation *in vitro* (Fig. 5J) and egression from the infected RBCs (Fig. S6C) and *P. berghei* ANKA infectivity in mice (Fig. 5K through M). These findings suggest that the 26S proteasome is critically involved in α-spectrin processing during the early stage of parasite development.

## DISCUSSION

Remodeling of the erythrocyte membrane and skeleton has been recognized as a critical process in the intraerythrocytic development of *P. falciparum* after infection (3, 14, 26), but the underlying mechanism remains elusive. In this study, we observed that the parasite selectively digests α-spectrin, which undermined the integrality of the EMS network. We further revealed a novel PfPI3K pathway that regulates EMS remodeling in *P. falciparum*. We observed that PfPI3K was expressed throughout the intraerythrocytic stages of the parasite (Fig. 1F; Fig. S2), which coincided with the selective degradation of erythrocytic α-spectrin but not β-spectrin. The degradation of host α-spectrin was partially blocked by PfPI3K inhibition (Fig. 3). Moreover, α-spectrin was predominantly ubiquitinated and degraded exclusively by the *P. falciparum* ubiquitin-26S proteasome complex, which could be blocked by ubiquitin ligases and 26S proteasome inhibitors (Fig. 4 and 5). Furthermore, we demonstrated that PfPI3K mediated the phosphorylation and activation of the E3 ubiquitin protein ligase NEDD4L, which in turn affects the recognition of ubiquitinated α-spectrin by the 26S proteasome (Fig. 4 and 5). In addition, inhibiting the PfPI3K-ubiquitin-proteasome pathway in iRBCs decreased parasite proliferation both *in vitro* and *in vivo* (Fig. 2, 4, and 5). However, previous studies have shown that the E3 ligases NEDD4 (27) and UBE3A (28) also contain a HECT domain. Heclin is likely to inhibit all three E3 ligases expressed during the intraerythrocytic cycle of the parasite. Even though NEDD4L was only found to be mainly located in the cytoplasm, we cannot completely rule out the involvement of NEDD4 and UBE3A in the processing of cytoskeletal proteins. The reason that blocking PfPI3K incompletely inhibited ubiquitination and α-spectrin degradation remains for further analysis. Additionally, investigation on the effect of the inhibitors of PfPI3K, the 26S proteasome, and the HECT domain of E3 ubiquitin ligases *in vivo* will provide more evidence of the importance of α-spectrin modification and processing for parasite development. Overall, this study provides evidence that *P. falciparum* parasites, through PfPI3K expression, thereby activated the ubiquitin protease-dependent pathway to destabilize the cytoskeleton network and ultimately enhance parasite development.

Ubiquitination is the primary mechanism underlying the selective degradation of EMS. Previous studies have reported that only α-spectrin undergoes ubiquitination in mature erythrocytes, whereas β-spectrin remains unmodified (13, 29, 30). Our results revealed that a higher level of ubiquitination on K2312 of α-spectrin was only observed in normal RBCs after *P. falciparum* invasion, and multiple lysine residues in α-spectrin were strikingly ubiquitinated, including K2281, K1441, K603, K628, K875, and K79 (Fig. 1G). However, LC-MS/MS identified no ubiquitination in β-spectrin (Fig. 1). Thus, selective ubiquitination is a prerequisite for *P. falciparum* development in erythrocytes after invasion.

Ubiquitin ligases have been well known to mediate the ubiquitination of substrate proteins (31). Here, three E3 ligases were predicted to interact with α-spectrin in iRBCs. Among them, only NEDD4L was located in the cytoplasm and showed detectable phosphorylation (Tables S3 and S4). We also found that PfPI3K phosphorylated and activated NEDD4L. Suppressing NEDD4L with its specific inhibitor, heclin, significantly inhibited α-spectrin ubiquitination after *P. falciparum* invasion. Consequently, α-spectrin degradation was markedly decreased, and importantly, parasite proliferation was significantly inhibited both *in vitro* and *in vivo* (Fig. 4J through M). Thus, the selective ubiquitination of α-spectrin by E3 ligases represents a critical process in parasite development.

The 26S proteasome recognizes and selectively degrades ubiquitinated proteins (21), and previous studies have identified traces of the 26S proteasome in mature erythrocytes (13, 14). Here, we observed a significant increase in 26S proteasome abundance in iRBCs upon *P. falciparum* invasion. Furthermore, suppression of the 26S proteasome using MG132 significantly inhibited α-spectrin degradation. These findings indicated that the 26S proteasome is a key enzyme responsible for the selective degradation of α-spectrin in iRBCs. Notably, studies have reported that the proteins of the scaffolds for α/β-spectrin heterodimer webs, including ankyrin, band 4.1, adducin 2, and dematin, are all degraded by the 20S proteasome rather than by the 26S proteasome (14), indicating that the processing of cytoskeletal proteins is differentially regulated.

**Fig 6:**
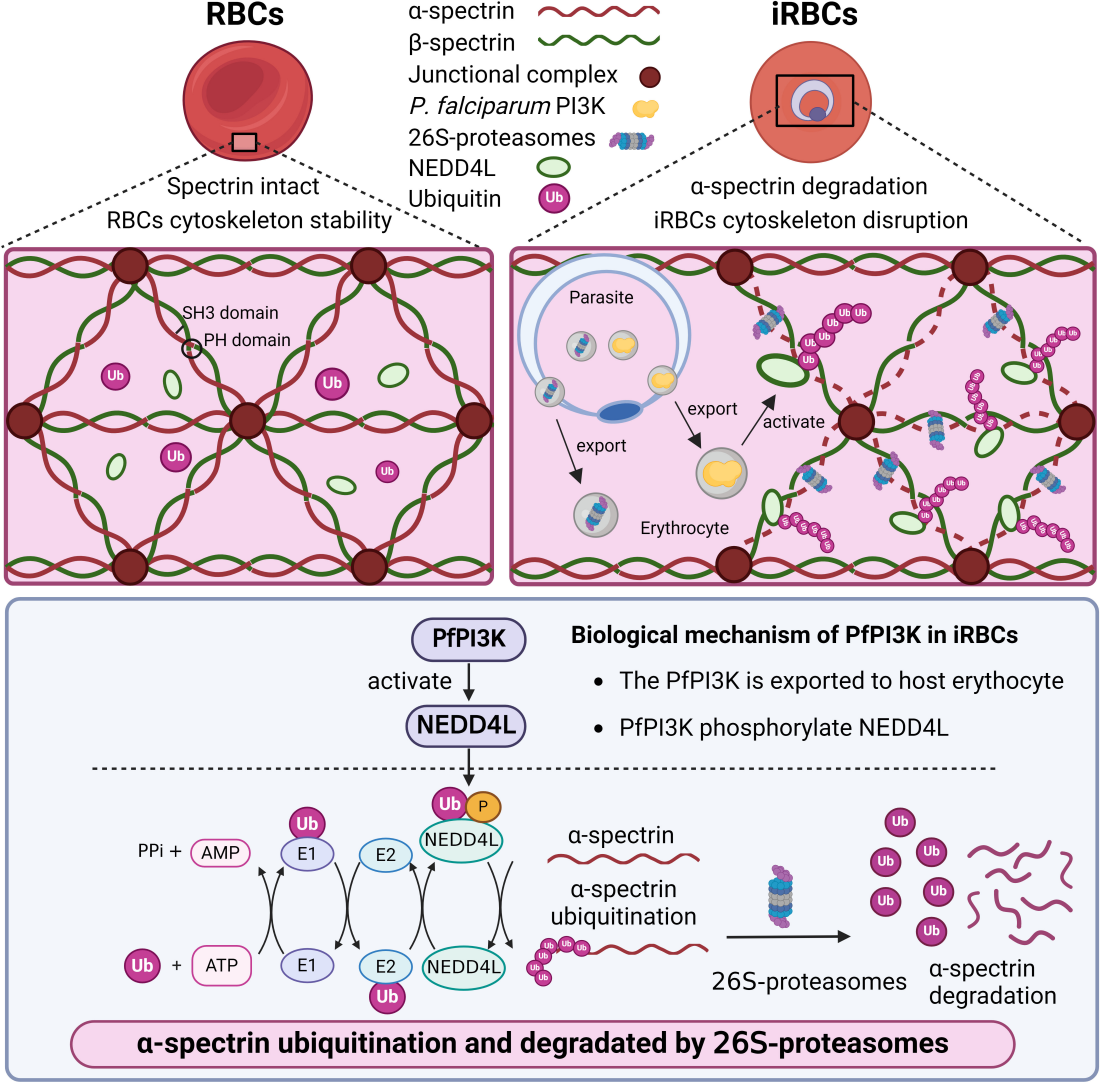
A schematic illustration of *P. falciparum* PI3K signaling in the regulation of selective α-spectrin processing. PfPI3K phosphorylates and activates *P. falciparum* ubiquitin-protein ligase, which promotes α-spectrin ubiquitination and degradation during the early stage of *P. falciparum* development.

It was known that PfPI3K phosphorylates phosphatidylinositol to generate phosphatidylinositol 3-phosphate, which is associated with the exportation of proteins from *P. falciparum* to the erythrocyte cytoplasm (18) and the transportation of hemoglobin from the erythrocytes to the food vacuole (20). Previous studies also suggested that dihydroartemisinin could suppress PfPI3K activity in the early developmental stage of *P. falciparum* (18, 32). We observed that inhibition of PfPI3K significantly impaired the ubiquitination and degradation of α-spectrin started at the ring stage of *P. falciparum*. Our data indicated that PfPI3K is a critical regulator of EMC remodeling through the activation of host E3 ligases (Fig. 6).

In conclusion, host α-spectrin is primarily ubiquitinated and degraded through E3 activity-dependent and proteasomal mechanisms after erythrocytic invasion by *P. falciparum*, which is regulated by PfPI3K signaling. This study provides a basis for elucidating the regulatory mechanisms involved in interactions between the host and malarial parasites.

## MATERIALS AND METHODS

### Mice

Female C57BL/6 mice aged 6–8 weeks were obtained from Changsheng Biological Technology Company (Benxi, Liaoning, China). The mice were housed in specific pathogen-free conditions and acclimatized for 1 week at a temperature of 18°C–22°C with a 12-h light/dark cycle before the start of the experiment. Throughout the study, mice were provided with regular rodent chow and had unrestricted access to clean drinking water.

### *P. falciparum* 3D7 culture and synchronization

*P. falciparum* 3D7 was cultured using standard methods with some modifications (16, 33). RBCs were washed twice with serum-free RPMI-1640 and stored at 4°C. Human serum was stored at −20°C until required. *P. falciparum* 3D7 parasites were cultured in human O^+^ RBCs at 5% hematocrit in a malaria culture medium, which contained 5% type A serum and 0.25% AlbuMAX II (Gibco, Invitrogen, New Zealand). The cultures were maintained in a cell incubator at 37°C with 5% CO_2_. Parasite growth was synchronized via successive rounds of treatment with Percoll and sorbitol (Sigma, St. Louis, MO, USA) to obtain a population of pure ring-stage, trophozoite-stage, and schizont-stage parasite-iRBCs, as previously reported (16, 33, 34).

### Determination of PfPI3K expression in the infected RBCs after *P. falciparum* invasion

The *P. falciparum* 3D7-iRBCs mixed cultures, which contained RBCs, ring-stage, trophozoite-stage, and schizont-stage parasite-iRBCs, were fixed on adhesive slides using pre-chilled methanol for 10 min. Cells were permeabilized and blocked with Immunol staining blocking buffer (containing Triton X-100, Beyotime, cat#P0102) at 37°C for 1 h and then incubated with the primary antibody rabbit anti-PIK3CG (Affinity BioReagents, cat#Ab-DF4764, 1:100) at 37°C for 1 h. Afterward, the slides were incubated with Alexa Fluor 488 goat anti-rabbit IgG (H + L) (Invitrogen, cat#2541675, 1:500) fluorescent secondary antibodies at 37°C for 30 min. Finally, the nuclei were stained with DAPI (Invitrogen, cat#2P36931). Images were captured using a Spinning Disk Confocal and Structured Illumination jointed Super-Resolution Microscope (SIM-ultimate, CSR Biotech Co., Ltd., Guangzhou, China).

### Drug inhibition of PfPI3K, E3 ubiquitin ligase, and 26S proteasome

*P. falciparum* strain 3D7 at the ring stage (6–10-h post-invasion) was used to test antimalarial effects in 96-well plates. Parasites were grown in O^+^ erythrocytes treated with 6, 12, 25, 50, or 100 µM of the PI3K inhibitors LY294002 (HY-10108, MCE, USA) or wortmannin (HY-10197, MCE, USA), or 5, 50, 500, 5, or 50 µM of GSK1059615 (HY-12036, MCE, USA) in up to three consecutive 72-h growth assays (20, 35). Ring-stage intraerythrocytic *P. falciparum* parasites were exposed to 1.5, 3, 6, 12, or 25 µM of the E3 ubiquitin ligase inhibitor heclin (HY-110204, MCE, USA) (36) or 10, 100, 1, 10, or 100 µM of the proteasome inhibitor MG132 (HY-13259, MCE, USA) for 72-h growth assays (37, 38). Next, whole-cell proteins were extracted from RBCs, iRBCs, and *P. falciparum* 3D7 cells after 6 h of treatment with specific inhibitors. Total RNA was extracted from *P. falciparum* 3D7 cells using the same inhibitor as described above.

For the *in vivo* drug inhibition studies, we used murine models infected with *P. berghei* ANKA or a transgenic *P. berghei* ANKA line expressing luciferase. C57BL/6 mice were randomly divided into four groups of 10 mice each, which were continuously analyzed on the same day. Each mouse received an intraperitoneal injection (i.p.) of 1 × 10^6^ parasitized RBCs. The next day, mice in the PI3K inhibitor group were treated with LY294002 (1 mg/kg of body weight) i.p. daily (39). In the E3 ligase inhibitor group, mice were treated with heclin (1 mg/kg of body weight) i.p. daily (40). In the 26S proteasome inhibitor group, mice were treated with MG132 (1.5 mg/kg of body weight) i.p. daily (41). In the control group, mice were treated with *i.p.* saline daily.

### Determination of parasitemia

For *in vitro* experiments*,* ring-stage iRBCs with 1.0% parasitemia were incubated with inhibitors (approximately 6–10-h post-invasion), as described above, and *P. falciparum* treated without the inhibitor was chosen as a control. Parasitemia at the next trophozoite stage (approximately 72-h post-invasion) was stained with Dihydroethidium (Sigma, cat#D7008) and monitored using a BD FACSAria III flow cytometer (BD Biosciences, USA) equipped with a 488 nm laser and a 610/20 nm optical filter (42). For *in vivo* experiments*, P. berghei* ANKA (1 × 10^6^) was injected i.p. into C57BL/6 mice, and parasitemia was monitored daily by regularly scoring the parasites in Giemsa-stained blood smears. Parasitemia was determined by counting the number of iRBCs (a minimum of three fields per blood smear) using a microscope (Leica Microsystems CMS GmbH DM4 B, Germany) at an objective lens magnification power of 100×. The percent parasitemia and inhibition were calculated using the modified Peters–Robinson formula (43–45).

### *In vivo* parasite imaging

*In vivo* imaging was used to quantify the prevalence of *P. berghei* ANKA-luc parasites in mice from different groups using an IVIS system (AniView600, Guangzhou Biolight Biotechnology Co., Ltd, Guangzhou, China). The mice were anesthetized and injected i.p. with 100 µL of D-luciferin potassium salt (15 mg/mL in PBS, Meilunbio, Cat. MB1834). After 2 min of incubation, the mice were placed in the IVIS system, and bioluminescence images were acquired using a medium binning factor and a field of view of 16.8 cm to capture the entire body. The exposure time for imaging ranged from 5 to 60 s. The data were analyzed using Living Imaging AniView software. All values represent the average of three measurements after subtracting the value of the blank measurement.

### Quantitative real-time RT-PCR

For drug inhibition experiments, total RNA was isolated from ring-stage iRBCs (6-h post-invasion) that had been subjected to inhibitor treatments for 6 h, as well as from the control group. Total RNA was isolated from different iRBC samples, including ring stage (8-h post-invasion), trophozoite stage (24-h post-invasion), and schizont stage (40-h post-invasion). RNA was isolated according to the manufacturer’s instructions using TRIZOL (Thermo Fisher, Carlsbad, USA). Subsequently, reverse transcription was conducted using the HiScript III RT SuperMix for qPCR (+gDNA wiper) Kit (Vazyme, Nanjing, China), and qPCR was performed using the SYBR Green MonAmp ChemoHS qPCR Mix kit (Monad, Suzhou, China) with primers corresponding to the sequences of PfPI3K (*P. falciparum*, PF3D7_0515300), proteasome subunit alpha type-7 (*P. falciparum*, PfPSMA1, PF3D7_1353900), 26S proteasome regulatory subunit RPN2 (*P. falciparum*, PfPSMD1, PF3D7_1466300), and glyceraldehyde-3-phosphate dehydrogenase (*P. falciparum*, PfGAPDH, PF3D7_1462800). Fluorescent signals were detected using an Applied Biosystems QuantStudio3 Real-Time PCR System (Thermo Fisher Scientific, USA). The 2^-ΔΔCT^ method was employed to determine the expression of the target genes *PfPI3K, PfPSMA1,* and *PfPSMD1* relative to *PfGAPDH*, an internal reference gene. The primer sequences used for qRT-PCR are listed in Table S1.

### Western blot assays

The *Plasmodium falciparum*-infected erythrocytes were lysed with 0.15% saponin to obtain the purified parasites. The iRBCs from both the experimental and control groups were collected and washed twice with 5 mL of PBS. A threefold volume of cell lysis solution, which contained phenylmethanesulfonyl fluoride (1 mM), phosphatase inhibitor (10 mM), and protease inhibitor (1 µM), was added to the purified parasites and cell precipitate. The purified parasites and cell lysates were sonicated in an ice-water bath using ultrasonic pulses (220 W, 3 s on/5 s off, 10 min). After centrifugation at 12,000 rpm, the supernatant was used to determine the protein concentration using a BCA kit (Sangon Biotech, cat#C503021). The protein solution was mixed with 5× loading buffer and denatured by boiling at 95°C for 10 min. Then, 20 µg of protein samples was separated using sodium dodecyl sulfate-polyacrylamide gel electrophoresis (SDS-PAGE, 10% or 6%) and transferred to a polyvinylidene difluoride membrane. Following protein transfer, the membrane was blocked with bull serum albumin (3%) and immunoblotted with different antibodies, including rabbit anti-PSMD1 (Affinity BioReagents, cat#Ab-DF13463, 1:1,000), rabbit anti-PSMA1 (PTM Biolabs, cat#PTM-5978, 1:1,000), rabbit anti-NEDD4 (Beyotime, cat#AF7554, 1:1,000), rabbit anti-NEDD4L (Affinity BioReagents, cat#Ab-AF4786, 1:1,000), rabbit anti-PIK3CG (Affinity BioReagents, cat#Ab-DF4764, 1:1,000), rabbit anti-GAPDH (Affinity BioReagents, cat#Ab-AF7021, 1:3,000), rabbit anti-phospho-NEDD4L(Ser342) (Affinity BioReagents, cat#Ab-AF4486, 1:1,000), rabbit anti-UBE2K (Affinity BioReagents, cat# Ab-DF6231, 1:1,000), rabbit anti-α-spectrin (BBI Life Sciences Corporation, cat#D163079, 1:1,000), rabbit anti-β-spectrin (BBI Life Sciences Corporation, cat#D163269, 1:1,000), rabbit anti-ubiquitin (PTM Biolabs, cat#PTM1106, 1:1,000), and anti-Plasmodium aldolase antibody (Abcam, cat#AB207494, 1:2,000) at 4°C overnight. After three washes, the membrane was incubated with HRP-conjugated goat anti-rabbit IgG secondary antibody (BBI Life Sciences Corporation, cat#D110058, 1:10,000) at 37°C for 1 h. After three washes, the proteins were visualized via enhanced chemiluminescence detection (Qinxiang ChemiScope 6200, China). Protein bands were quantified using ImageJ software (NIH, USA) and an e-BLOT touch imager (e-BLOT Life Science, Shanghai, China). All experiments were repeated with three biological samples.

### Immunofluorescence staining of α-spectrin and β-spectrin

Immunofluorescence staining was performed on iRBCs following a previously described protocol with the following modifications (20). *P. falciparum* 3D7-iRBCs were fixed on adhesive slides using pre-chilled methanol for 10 min. Parasites were permeabilized with 0.05% Triton X-100 at room temperature for 10 min. First, the slides were blocked with 5% BSA at 37°C for 1 h and then incubated with the primary antibodies rabbit anti-α-spectrin (BBI Life Sciences Corporation, cat#D163079, 1:500) and rabbit anti-β-spectrin (BBI Life Sciences Corporation, cat#D163269, 1:100) at 37°C for 1 h. Afterward, the slides were incubated with Alexa Fluor 488 goat anti-rabbit IgG (H + L) (Invitrogen, cat#2541675, 1:500) fluorescent secondary antibodies at 37°C for 30 min. Finally, the nuclei were stained with DAPI (Invitrogen, cat#2P36931). Images were captured using a confocal laser scanning microscope (Leica SP8, Wetzlar, Germany).

### Co-IP with anti-α-spectrin and anti-ubiquitin antibodies

Total protein was extracted from normal RBCs for α-spectrin and ubiquitin co-IP. RBCs were treated with co-IP lysis buffer (25 mM Tris-HCl, 150 mM NaCl, 1 mM EDTA, and 1% NP-40; Servicebio, cat#G2038) containing a protease inhibitor cocktail (Servicebio, cat#G2006). The lysate was clarified via centrifugation (12,000 rpm, 10 min, 4°C), and total protein concentration was measured using Nanodrop-2000C (Thermo Fisher Scientific, USA). The cell protein (500 µg) was incubated with mouse anti-α-spectrin monoclonal antibody (1 µg, Abnova, cat#MAB2515) overnight rotating at 4°C, and mouse IgG (1 µg, Solarbio, cat#SP031) was used as a control. Next, 50% protein G agarose beads (50 µL, Cytiva, cat#17061801) were added to the cell protein mix and incubated while rotating for 3 h at 4°C. Following incubation, agarose beads were washed five times with 1 mL of co-IP lysis buffer and eluted with 15 µL of 5× SDS sample loading buffer (Beyotime, cat#P0015L) boiled for 10 min. Proteins were released, separated on 6% SDS-PAGE, immunoblotted using primary antibodies with mouse anti-α-spectrin (Abnova, cat#MAB2515, 1:1,000) and rabbit anti-ubiquitin (PTM Biolabs, cat#PTM1106, 1:1,000), and then incubated with the differentially labeled species-specific secondary antibodies, HRP-conjugated goat anti-rabbit IgG (BBI Life Sciences Corporation, cat#D110058, 1:10,000) and HRP-conjugated goat anti-mouse IgG (BBI Life Sciences Corporation, cat#D110087, 1:10,000). Protein bands were visualized using a chemiluminescence imaging system (Qinxiang ChemiScope 6200, CA, USA).

### Detection of PfPI3K inhibitory effect of ring stage iRBCs through proteomic analysis

#### Experimental design and statistical rationale

The global proteome of soluble proteins derived from ring-stage iRBCs was analyzed using 4D label-free mass spectrometry (Novogene Co. Ltd.). The control and PfPI3K inhibitor-treated groups (treated with 50 µM LY294002 and incubated for 6 h) with three biological replicates were included in the analysis. Qualitative and quantitative analyses were performed for the different treatment groups. A standard *t*-test was conducted to determine whether there was a statistically significant difference between the two treatment groups. Statistical testing and graph generation were performed using R software.

#### Protein extraction and trypsin digestion

The iRBCs were washed with pre-cooled PBS. Samples in protein lysis buffer (8 M urea and 100 mM TEAB, pH = 8.5) were sonicated (220 W, 3 s, interval of 5 s for 5 min) on ice and then centrifuged at 12,000 rpm at 4°C for 15 min. The supernatants were collected, and DTT (10 mM) was added for 1 h at 56°C. Next, the samples were treated with iodoacetamide (Sigma, cat#I6125-25G) and incubated at room temperature for 1 h in the dark. Protein concentration was determined using a Bradford protein quantitation kit. The quality of the protein samples was determined using SDS-PAGE.

Before trypsinization, protein lysis buffer (8 M urea and 100 mM TEAB, pH = 8.5) was added to the appropriate amount of protein solution to a final volume of 100 µL. Trypsin and TEAB buffer (100 mM) were added, and the mixture was digested at 37°C for 4 h. Trypsin and CaCl_2_ were added to the samples for overnight digestion. Trypsin-digested peptides were desalted using C18 desalting columns. The column was washed three times with a cleaning solution (0.1% formic acid and 3% acetonitrile). Trypsin-digested peptides were eluted and lyophilized.

#### Ultra-high-performance chromatography fractionation and LC-MS/MS analysis of the proteome

The tryptic peptides were dissolved in solvent A (0.1% formic acid and 100% water). The gradient of solvent B (0.1% formic acid in 100% acetonitrile) was increased from 2% to 22% (45 min), 22% to 35% (5 min), and increased to 80% (5 min), then held at 80% for the last 3 min, at a constant flow rate of 300 nL/min on a nanoElute-upgrade ultra-high-performance chromatography (UHPLC) system (Bruker, Germany). The peptides were subjected to a nano-spray ion source, followed by MS/MS in a timsTOF pro2 (Bruker, Germany) coupled online to a UHPLC. The applied electrospray voltage was 1.5 kV. The *m/z* scan range was 100–1,700 for full scans. The 1/K0 range was 0.85–1.3 V s/cm^2^, ramp time was 100 ms, the lock duty cycle was set to 100%, and PASEF settings were 10 MS/MS scans (the total cycle time was 1.17 s). The target ion intensity was set to 20,000, with an ion intensity threshold of 2,500.

### Bioinformatics analysis

Quantitative proteomic, quantitative ubiquitinomic, and quantitative phosphoproteomic analyses were performed as previously described (16). KEGG enrichment analysis was performed using the ClusterProfiler package. Heatmaps depicting the ubiquitin modification sites of α-spectrin and β-spectrin were generated using TBtools. Statistical analyses were performed on phosphorylated E3 ligases. The E3 ubiquitin ligases that interact with α-spectrin were predicted using the http://ubibrowser.bio-it.cn/ website.

### Statistical analysis

Data were collected from independently repeated experiments (*N* ≥ 3) and analyzed using GraphPad Prism 10 software. The statistical significance of all differences was determined using a two-tailed *t*-test or one-way analysis of variance with the Bonferroni *post hoc* test.

## SUPPLEMENTAL MATERIAL

Supplemental figures mbio.03510-23-s0001.docx Figures S1 to S6.

Table S1 mbio.03510-23-s0002.docx Primers used in this study.

Table S2 mbio.03510-23-s0003.xls Ubiquitination sites identified in spectrin alpha chain.

Table S3 mbio.03510-23-s0004.xls E3 ubiquitin-protein ligases identified in the proteomic analysis.

Table S4 mbio.03510-23-s0005.xls Phosphorylated E3 ubiquitin-protein ligase identified in the proteomic analysis.

Table S5 mbio.03510-23-s0006.xls 20S proteasome identified in the proteomic analysis.

Table S6 mbio.03510-23-s0007.xls 26S proteasome identified in the proteomic analysis.

ASM does not own the copyrights to Supplemental Material that may be linked to, or accessed through, an article. The authors have granted ASM a non-exclusive, world-wide license to publish the Supplemental Material files. Please contact the corresponding author directly for reuse.
