# Supplementary material for: Plasmodium falciparum selectively degrades α-spectrin of infected erythrocytes after invasion
Source: mBio. 2024 Mar 12;15(4):e03510-23. doi: 10.1128/mbio.03510-23 (PMC11005373; doi:10.1128/mbio.03510-23)
Supplement: Supplemental figures — Figures S1 to S6. [file mbio.03510-23-s0001.docx]

**Supplementary Figures:**


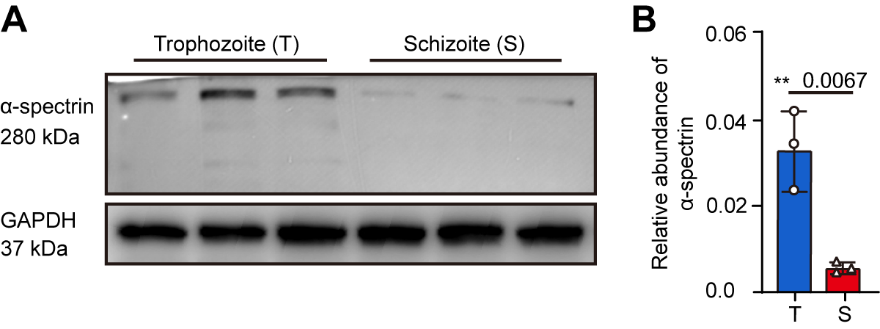


**FIG S1.** α-Spectrin is predominantly degraded in iRBCs after *P. falciparum* invasion. **(A)** Western blots of α-spectrin with anti-α-spectrin antibodies in the trophozoite and schizont stages of *P. falciparum*-iRBCs shown using the e-BLOT touch imager. **(B)** Relative quantification of α-spectrin protein levels (Figure S1A).


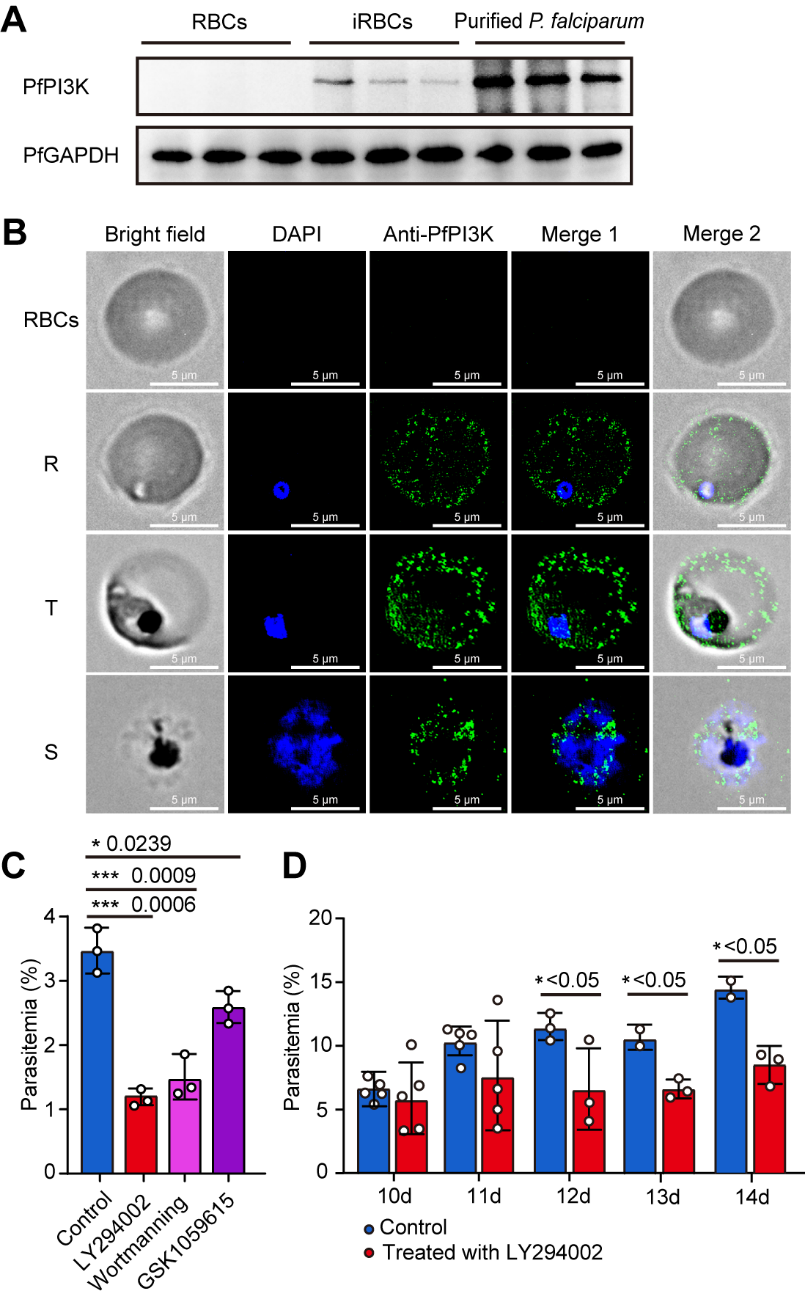
**FIG S2.** PfPI3K is essential for infection of blood-stage *Plasmodium* parasites. **(A)** Western blots of PfPI3K with anti-PfPI3K antibodies in the RBCs, iRBCs and purified *P. falciparum.* **(B)** PfPI3K was detected via immunofluorescence staining of RBCs and iRBCs using an anti-PI3K antibody. **(C)** Parasitemia of *P.* *falciparum* 3D7 was significantly inhibited by the PfPI3K inhibitors LY294002 (50 μM), wortmannin (50 μM), and GSK1059615 (10 μM). **(D)** LY294002 treatment significantly reduced parasitemia in *P. berghei* ANKA-infected mice over 14 days.


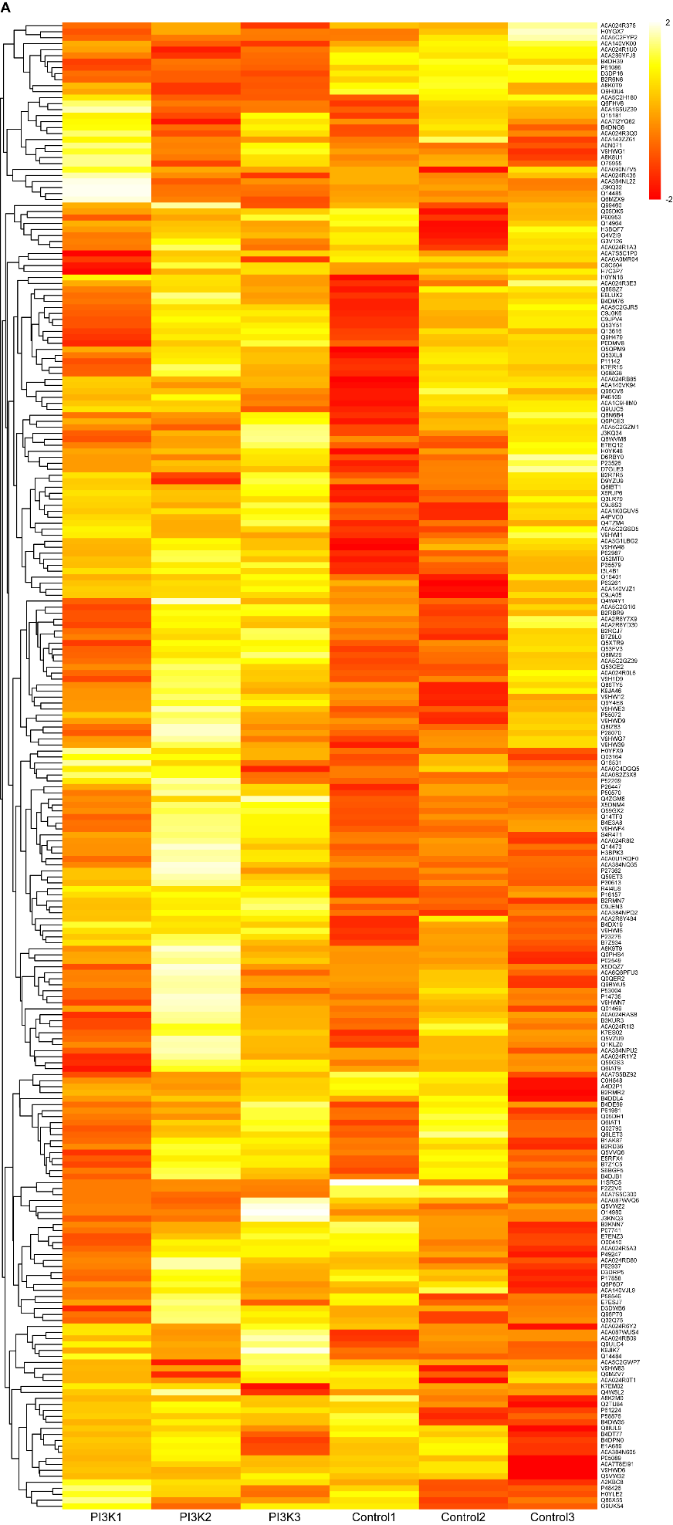


**FIG S3.** The heatmap of differential expression of genes of differentially expressed genes of ring-stage *P. falciparum* 3D7 strain with or without treatment of PfPI3K inhibitor LY294002.


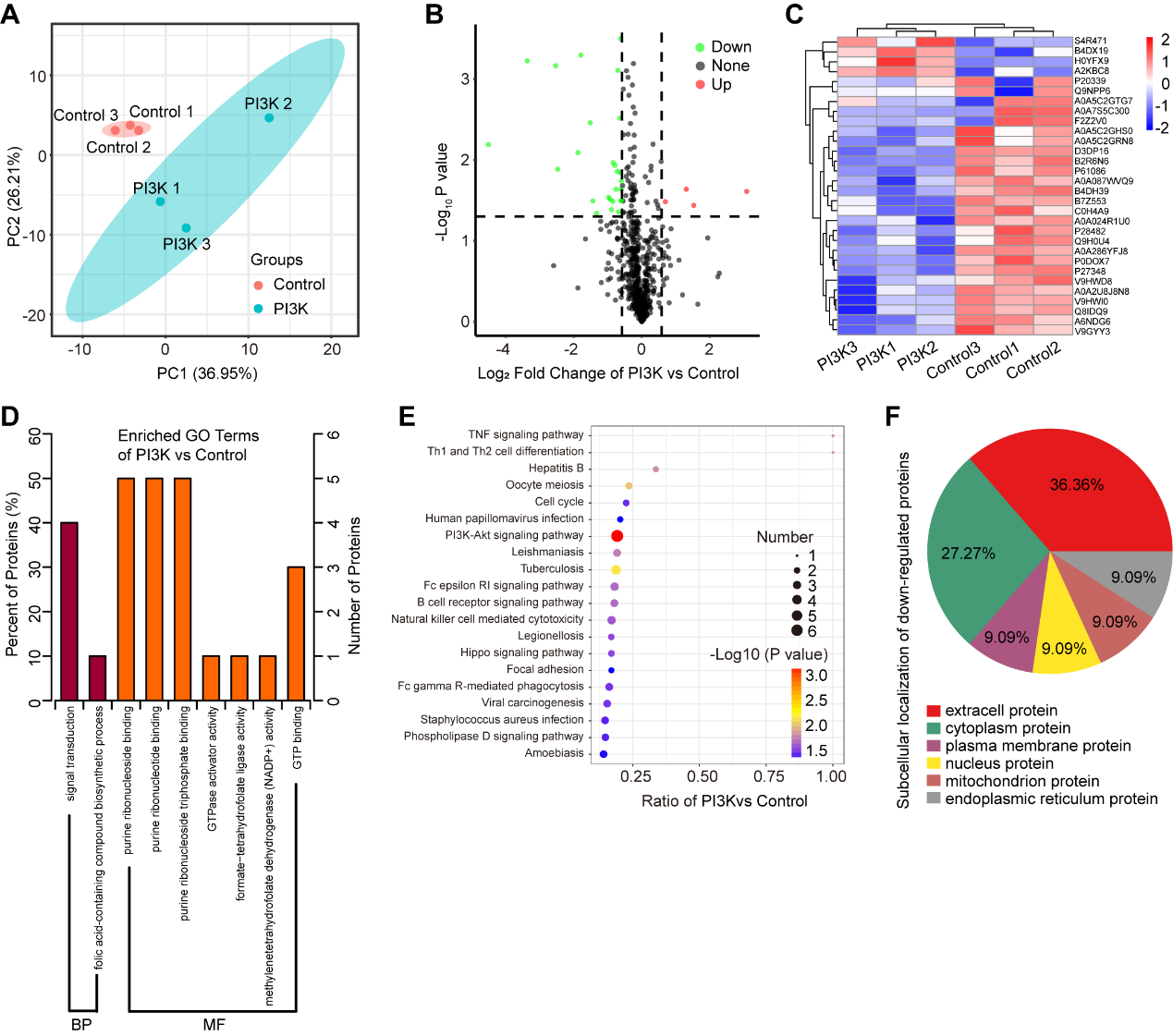


**F****IG S4.** Quantitative proteomic analysis of ring-stage *P. falciparum* 3D7 strain and treatment with the PfPI3K inhibitor LY294002. **(A)** Principal component analysis showed good duplication within groups and significant differences between groups. The PI3K groups represented the iRBCs incubated with LY294002. **(B)** Volcano plot showing differential protein expression in ring-stage iRBCs between the control and PfPI3K inhibitor-treated groups. **(C)** Cluster analysis of the relative content of differentially expressed proteins in each group. **(D)** Gene ontology enrichment result of differentially downregulated proteins. **(E)** KEGG pathway enrichment results for differentially downregulated proteins. **(F)** Subcellular localization analysis of the differentially downregulated proteins.


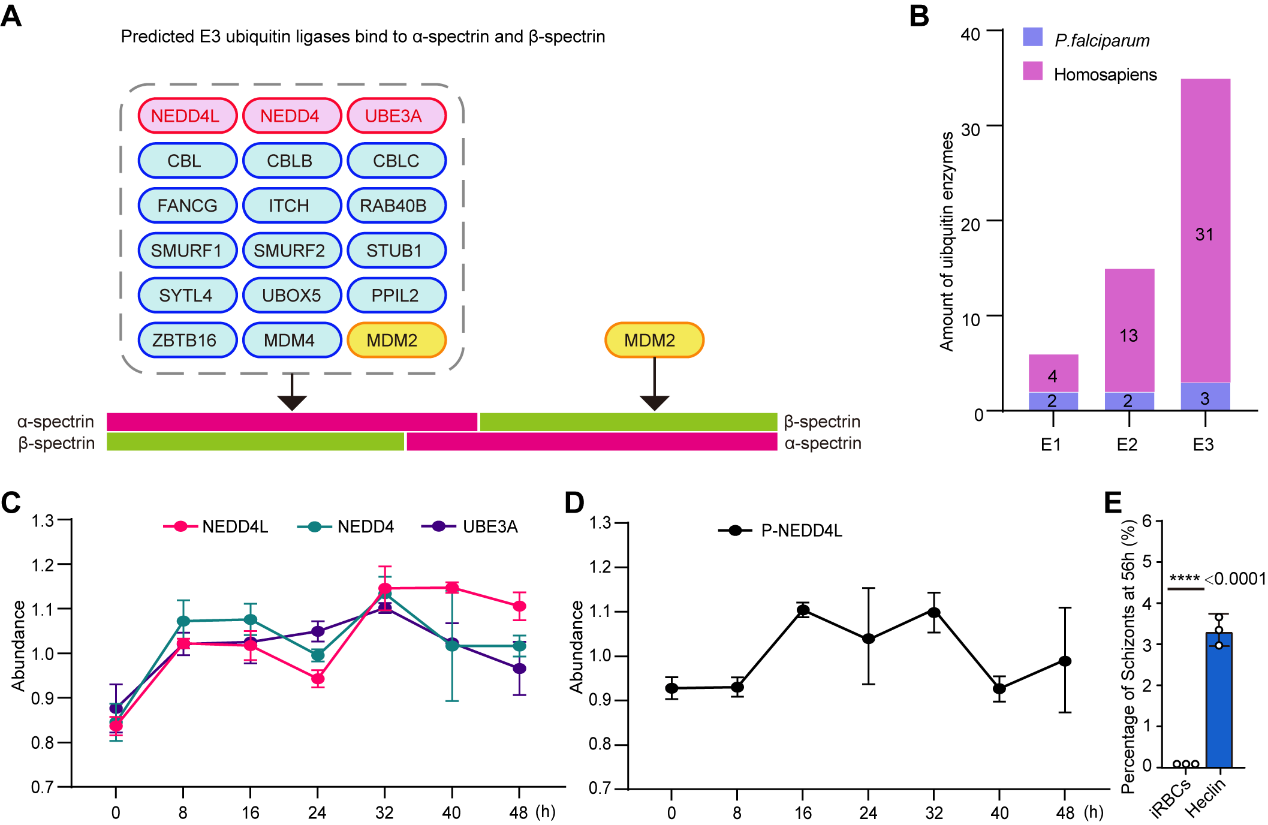


**FIG S5.** Prediction and screening of host α-spectrin E3 ligase and detection of its inhibitory effect on parasite egression from the infected RBCs. **(A)** E3 ubiquitin ligase binding to α-spectrin and β-spectrin was predicted using a software (<http://ubibrowser.bio-it.cn/>). **(B)** Proteomic identification of E3 ubiquitin ligases in humans and *P.* *falciparum* 3D7. **(C)** Analysis of the abundance of E3 ubiquitin ligases NEDD4L, NEDD4, and UBE3A during *P.* *falciparum* 3D7 parasite development at the blood stage. **(D)** Abundance of phosphorylated E3 ubiquitin ligase P-NEDD4L during *P.* *falciparum* 3D7 parasite development in the blood. **(E)** *P.* *falciparum* 3D7 parasitemia under treatment with NEDD4L inhibitor Heclin (5 μM) showed delayed egression from the infected RBCs.


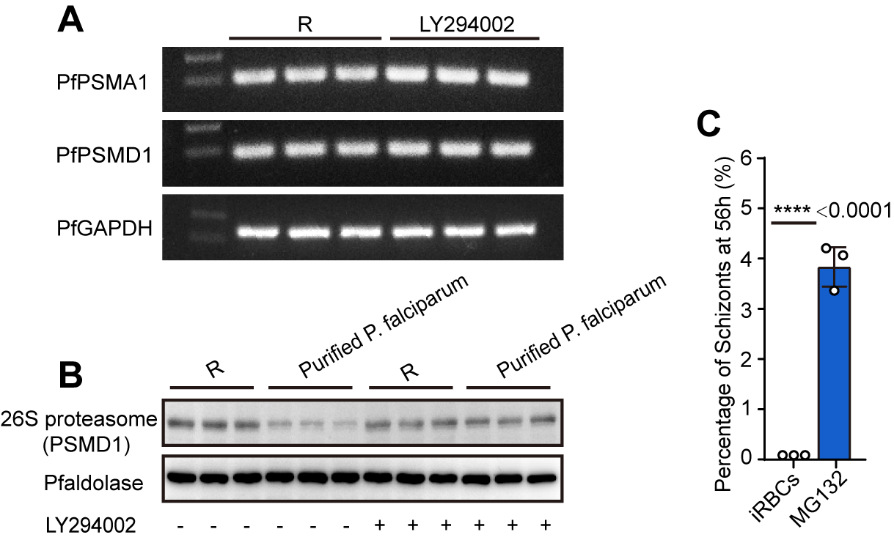


**FIG S6.** The transcription, expression and secretion of 26S proteasome in blood-stage *Plasmodium* parasites and detection of its inhibitory effect on parasite egression from the infected RBCs. **(A)** The PfPI3K inhibitor LY294002 did not inhibit the transcription of PfPSMA1 (*P.* *falciparum* 20S proteasome) or PfPSMD1 (*P.* *falciparum* 26S proteasome) in the ring stage of *P.* *falciparum* 3D7 parasites, as determined via RT-PCR. **(B)** The PfPI3K inhibitor LY294002 suppressed the secretion of 26S proteasome from the parasite to the erythrocyte cytoplasm. **(C)** *P.* *falciparum* 3D7 parasitemia under treatment with 26S proteasome inhibitor MG132 (100 nM) showed an inhibitory effect on parasite egression from the infected RBCs.
