# Supplementary material for: Plasmodium falciparum selectively degrades α-spectrin of infected erythrocytes after invasion
Source: mBio. 2024 Mar 12;15(4):e03510-23. doi: 10.1128/mbio.03510-23 (PMC11005373; doi:10.1128/mbio.03510-23)
Supplement: Table S1 — Primers used in this study. [file mbio.03510-23-s0002.docx]

| **Table S1** Primers used in this study | |
| --- | --- |
| ***Name*** | ***Sequence*** |
| PfPI3K-F | TGCCGTCACATGATAAAATGATGCC |
| PfPI3K-R | CCTAACGAGGAAACGCCTGATGG |
| PfPSMA1-F | GCCTTTGCTGGTTTAAATGCTG |
| PfPSMA1-R | ATCTACAGGTGCCGGTTCATC |
| PfPSMD1-F | CGCCTTTAGAACCATCAGAATGTATTG |
| PfPSMD1-R | GGTTCAAAGGGTGGTGCCTC |
| PfGAPDH-F | CGGACGTTTAGTATTTAGAGCAGCC |
| PfGAPDH-R | TCAGCGTGGGTTACCTCACA |
